# Structural Characterization of *Gracilariopsis lemaneiformis* Polysaccharide and Its Property in Delaying Cellular Senescence

**DOI:** 10.3389/fnut.2022.876992

**Published:** 2022-05-16

**Authors:** Xiaomei Wang, Xiaogang Xu, Genxiang Mao, Yue Guo, Guangce Wang, Xue Sun, Nianjun Xu, Zhongshan Zhang

**Affiliations:** ^1^Key Laboratory of Vector Biology and Pathogen Control of Zhejiang Province, Huzhou University, Huzhou, China; ^2^Key Lab of Geriatrics & Geriatrics Institute of Zhejiang Province, Department of Geriatrics, Zhejiang Hospital, Hangzhou, China; ^3^CAS and Shandong Province Key Laboratory of Experimental Marine Biology, Center for Ocean Mega-Science, Institute of Oceanology, Chinese Academy of Sciences, Qingdao, China; ^4^National Engineering Research Laboratory of Marine Biotechnology and Engineering, Key Laboratory of Aquacultural Biotechnology, Ministry of Education, Collaborative Innovation Center for Zhejiang Marine High-Efficiency and Healthy Aquaculture, Key Laboratory of Marine Biotechnology of Zhejiang Province, Ningbo University, Ningbo, China

**Keywords:** *G. lemaneiformis*, polysaccharide, cellular senescence, SA-β-gal, p53-p21 pathway

## Abstract

The sulfated polysaccharide was isolated from the purified *G. lemaneiformis* polysaccharide (PGP), and its property in delaying H_2_O_2_-induced 2BS cellular senescence was studied. The results showed that PGP was a linear polysaccharide containing alternating α-(1 → 3)- and β-(1 → 4)-galactopyranose units. Most of the sulfate groups are at C6 of the -(1 → 4)-α-D-Galp, and a small part of them are at C3 and C6. PGP pretreatment could decrease SA-β-gal-positive cells and prevent the formation of senescence-associated heterochromatic foci (SAHF) induced by H_2_O_2_ in a dose-dependent manner. It is speculated that PGP may delay aging by downregulating the expression of p21 and p53 genes. The finding provides new insights into the beneficial role of *G*. *lemaneiformis* polysaccharide (GP) on retarding senescence process.

## Introduction

Cellular senescence is a physiological stress response of mammalian cells which results in the development of senescent cells with distinct physical, molecular, and metabolic signatures (1). In fact, aging is a key cellular program that can be induced and plays an important role in permanently limiting the reproduction of damaged and defective cells (2). Human embryonic fibroblasts can be cultured *in vitro* for about 50 generations. With the increase of generations, cell division and proliferation are slow and gradually show the characteristics of cell aging. The main result is that the cell volume increases, cell heterogeneity increases, and chromosome aberration and lysosome increase (3). Growth arrest is not only a prominent manifestation of cell aging but also an important factor causing biological aging. Therefore, the aging response can be prematurely induced in cells through the application of endogenous and exogenous stimuli *in vitro* and *in vivo*. H_2_O_2_ has been the most commonly used inducer for stress-induced premature senescence, which shares the features of replicative senescence (4). Oxidative stress-induced premature aging has been a good tool for *in vitro* aging research.

*Gracilariopsis lemaneiformis*, a kind of delicious seafood, is rich in active ingredients, including vitamins, polysaccharides, nicotinic acid, proteins, and dietary fiber (5). It was indicated that *G*. *lemaneiformis* polysaccharides (GPs) possess various biological activities, such as antitumor, antioxidant, and immune regulatory (6–11). Till present, the primary method of polysaccharide extraction is using hot water. However, there is a disadvantage of high energy consumption and long extraction time (12). Acid can effectively degrade the glycosidic bonds of polysaccharides and improve the yield of small molecular polysaccharides (13). Hydrochloric acid and citric acid are the most commonly used as medium at present.

In this study, a model *in vitro* was established that the human embryonic lung diploid cells (2BS) were treated with a sub-lethal dosage of H_2_O_2_ to study the protective potential of PGP against cellular senescence. Alterations in cellular morphology, senescence-associated β-galactosidase (SA-β-gal) activity, cell cycle, and molecular expression were evaluated to illustrate the possibly associated molecular mechanisms.

## Materials and Methods

### Samples

*G*. *lemaneiformis* was collected from Xiapu County farm, Fujian Province. It was cleaned repeatedly and removed sediment and other sundries in the laboratory, then grounded and crushed after natural drying, and sealed for standby after 200 mesh screening.

### Extraction and Purification of GP

A solution of 50 g seaweed powder in 1,000 ml distilled water was added to 0.15 g cellulose and stirred at 50°C for 3 h. Later, 25 g citric acid monohydrate was added for reaction at 95°C for 2 h. The extraction solution was filtered and centrifuged at 5,000 r/min for 15 min. The supernatant was adjusted to pH 7 with 10% ammonia and then concentrated and dialyzed. The extract solution was added to ethanol, and the precipitate was dried to obtain GP.

A solution of GP in distilled water was purified by gel chromatography on a Sephadex G-100 column (50 cm × 2.6 cm) eluted with tap water (0.2 ml/min). The fraction obtained was dialyzed and freeze-dried to yield a purified polysaccharide (PGP).

### Structural Analysis

Routine component, including total sugar and sulfate content, was detected according to the previous method (14–16). Infrared spectrums were measured by a Nicolet Avatar 360 with KBr disks. Molecular weight and monosaccharide composition were determined by High Performance Gel Permeation Chromatography (HPGPC) and Gas Chromatography—Mass Spectrometry (GC-MS). ^1^H and ^13^C NMR spectra (e.g., 2D COSY, TOCSY, and DEPT) of samples were recorded using an AVANCE 600 MHz with a triple resonance probe.

### Cell Viability Assay

2BS cells were cultured as a monolayer in Dulbeo's Modification of Eagle's Medium (DMEM) supplemented with 10% Fetal Bovine Serum (FBS) in a 37°C, 5% CO_2_ humidified incubator. For drug administration, the culture medium was removed and PGP at indicated concentrations (e.g., 10, 50, 100, and 200 μg/ml) was added into each well for 2 h incubation, followed by treatment with 200 μM H_2_O_2_ for 1 h to induce premature senescence. Then, the H_2_O_2_-containing medium was replaced with a fresh medium for an additional 48 h before harvest. Cell viability was assessed by the Cell Counting Kit-8 assay, as described previously (17). Each compound was tested in triplicate and the experiments were repeated three times.

### SA-β-Gal and DAPI Staining

Detection of *SA-*β*-Gal*-positive cells was performed, as described previously, according to the indications provided with the staining kit (18). All the experiments were repeated three times, and one of the representative results was shown. DNA was visualized by 4',6-diamidino-2-phenylindole (DAPI) (1 μg/ml) after fixation with 4.0% paraformaldehyde (PFA) (19).

### Western Blot Analysis

An equal number of 2BS cells from the different groups were lysed using a sample buffer. Western blotting was performed, as described previously, according to the standard procedures. The sample was separated by 10% Sodium Dodecyl Sulfate PolyAcrylamide Gel Electrophoresis (SDS-PAGE) and transferred to nitrocellulose membranes. After being blocked in evaporated non-fat milk for 4 h, the membranes were incubated with 1% Bovine Serum Albumin (BSA) solution dissolving primary anti-bodies (p53, p21). Protein bands were visualized using horseradish peroxidase-conjugated secondary antibodies. Protein bands were quantified by densitometry and normalized to glyceraldehyde-3-phosphate dehydrogenase (GAPDH).

### Statistical Analysis

All the data were presented as mean ± SD. A value of *p* < 0.05 was considered statistically significant. Differences between groups were evaluated by one-way ANOVA followed by Duncan's multiple range tests.

## Results

### Chemistry of PGP

**Figure 1:**
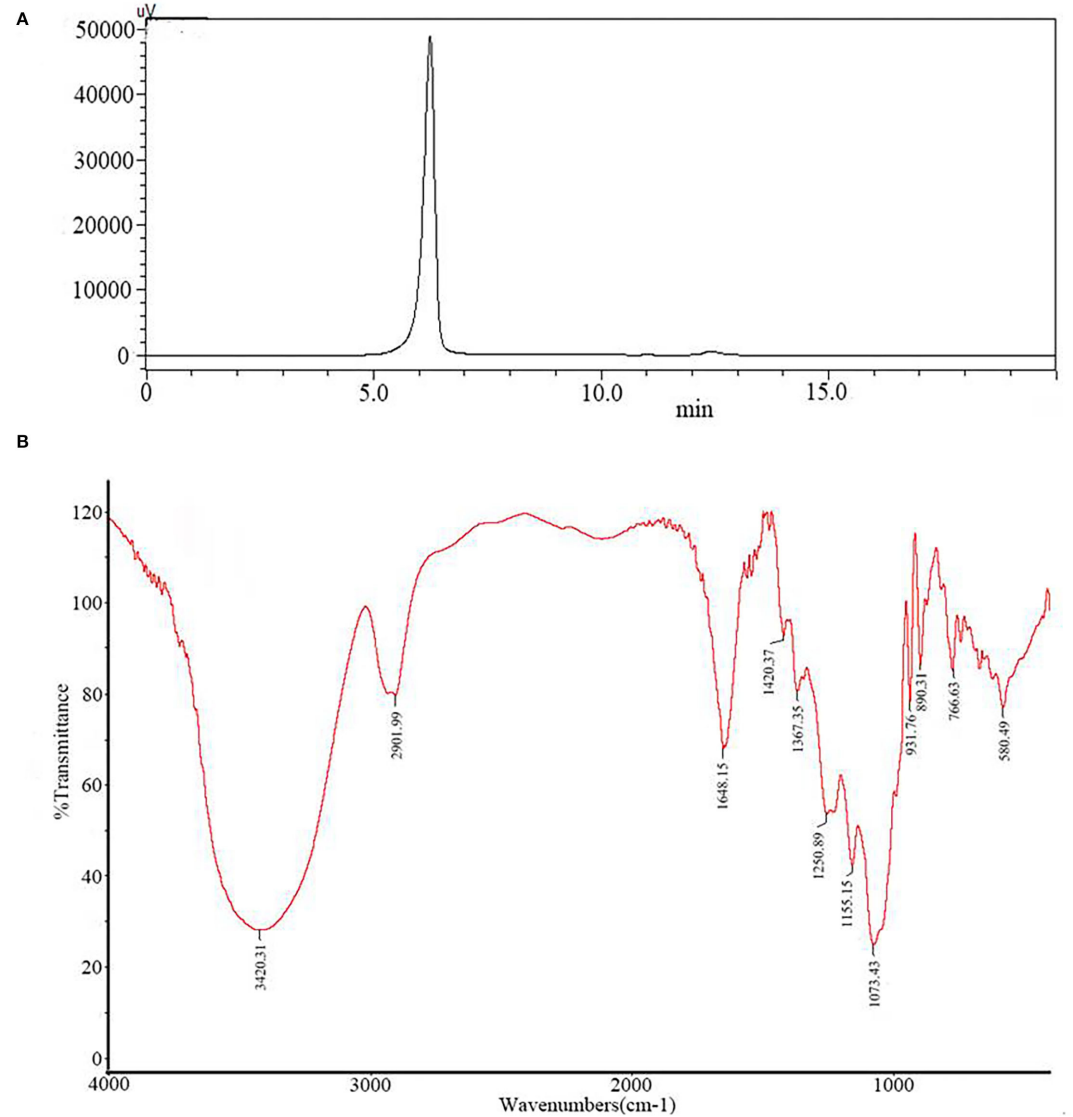
The molecular weight distribution **(A)** and infrared spectrum **(B)** of PGP.

The soluble polysaccharide of *G*. *lemaneiformis* (GP) obtained by acid extraction was a non-gelling polysaccharide. Gel chromatography on dextran G100 separated the sulfated polysaccharide from GP into one peak, named PGP (Figure 1A). The latter was eluted as a single symmetrical peak corresponding to an average molecular weight of 2.7 × 10^3^ Da, as determined by the high Performance Liquid Chromatography (HPLC) method. A lack of absorption at 280 nm indicated that PGP contained no protein.

The PGP contained 92.65% of total sugar and 5.37% of sulfate on the basis of fraction dry weight. Sugar compositional analysis revealed that PGP consists of polysaccharides containing a high amount of galactose together with a smaller amount of glucose unit.

Figure 1B exhibits the major functional groups and the chemical bonds of PGP. As shown, the broad absorption at 3,420 cm^−1^ was corresponded to the O–H stretching vibration, and the strong band at 2,902 cm^−1^ was ascribed to stretching vibrations of C–H. Two bands at 1,648 and 1,420 cm^−1^ were assigned to the absorbance of the deprotonated COO– group (20). These characteristics indicated that PGP was a typical acidic polysaccharide, which agreed with the content of uronic acid (9.84%). The bands at 1,250 and 931 cm^−1^ can be attributed to the S=O vibration of the sulfate groups and the C–O–C of 3,6-anhydro-a-L-galp (21). The peak at 820 cm^−1^ indicated that sulfate groups were located in position C6 of the galactose ring (22).

### Structure of PGP

Methylation analysis of the sulfated PGP demonstrated the presence of a variety of methylated derivatives. In fact, methylation of sulfated polysaccharides does not always yield reliable proportions of methylated alditol acetates (23, 24). As shown in Table 1, it was probable that there was incomplete methylation. The peak type was relatively simple. The terminal group only exists in the form of Gal-(1 → indicating that galactose is the main terminal residue of PGP. The absence of other branched end residues indicated that most of the branched chains may be degraded by citric acid. The proportion of 1 → 3 Gal and 1 → 4 Gal was similar, indicating that there was a common disaccharide unit in red algae. → 3,6)-Galp-(1 → indicated that there were substituted sulfate and galactose residues at C-6.

**Table 1:** Analysis of PGP methylation.

**RT**	**Methylated sugar**	**Mass fragments (m/z)**	**Molar ratios**	**Type of linkage**
17.661	2,3,4,6-Me_4_-Galp	43,71,87,101,117,129,145,161,205	0.10	Gal-(1 →
21.247	2,3,6-Me_3_-Galp	43,87,99,101,113,117,129,131,161,173,233	0.34	→ 4)-Galp-(1 →
21.549	2,4,6-Me_3_-Galp	43,71,85,87,99,101,117,129,161	0.42	→ 3)-Galp-(1 →
22.362	2,4-Me_2_-Galp	43,71,87,99,101,117,129,139,159,173,189	0.14	→ 3,6)-Galp-(1 →

**Figure 2:**
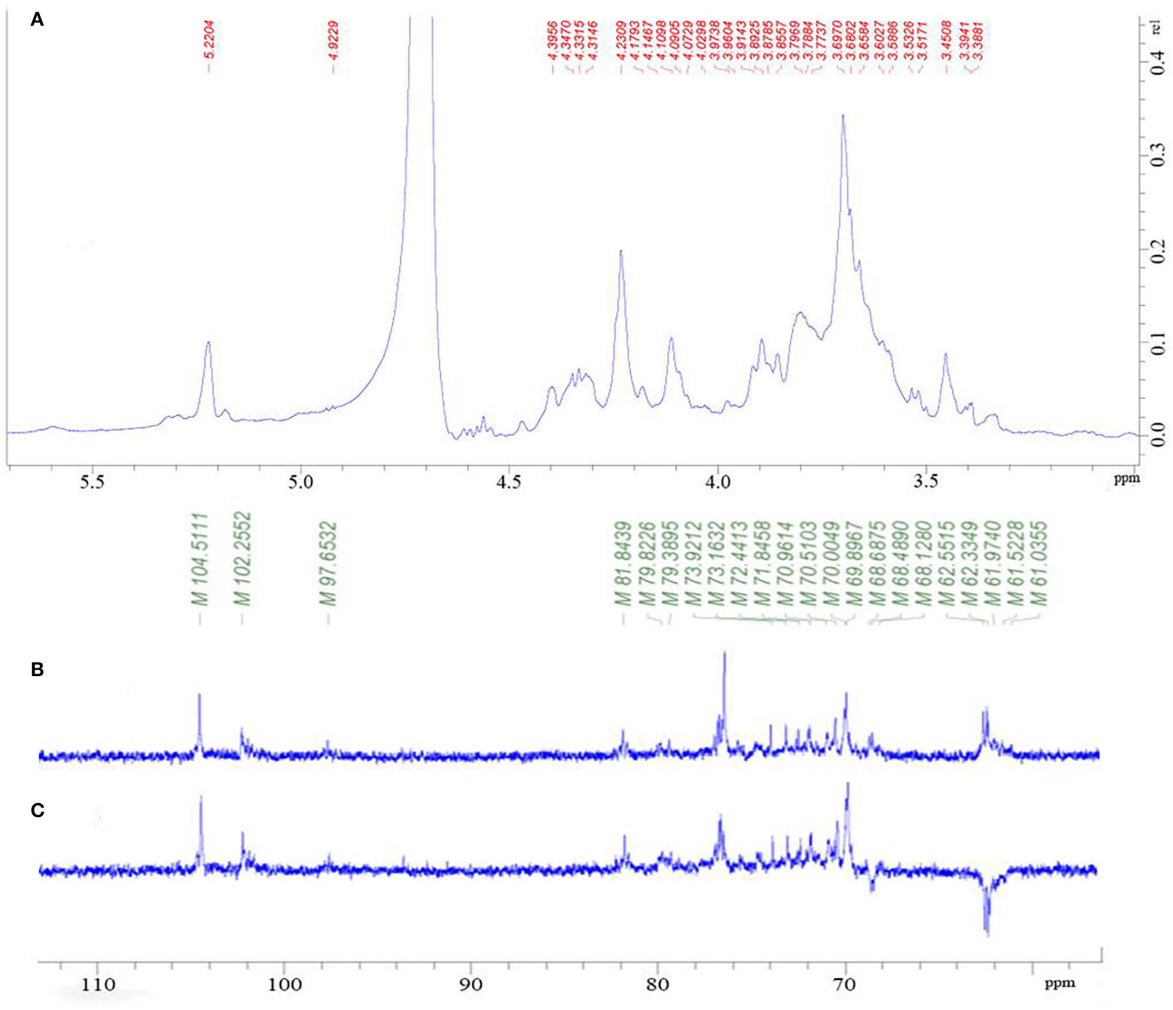
^1^H NMR spectrum of PGP **(A)**; ^13^C-NMR spectrum of PGP **(B)**; DEPT135 spectrum **(C)** of PGP.

The NMR analysis was employed to determine the saccharide backbone structure and also to determine the sulfation pattern of the sulfated galactan. From Figure 2A, the proton signal of the sugar ring is mainly concentrated in δ 3.2–4.0. Furthermore, the signal peaks of end group proton (δ 5.22, 4.92, 4.40, and 4.35) were concentrated in the area of δ 4.3–5.5. The signals from an anomeric proton at δ 5.15 and 5.22 were assigned to 3,6-α-L-anhydrogalactose (LA) and α-L-galactose-6-sulfate (L-6S), respectively. However, it was obvious that the signal of δ 5.22 was much greater than δ 5.15, indicating the C-6 position of (1 → 4)-α-L-galactose was mainly substituted by the sulfate group but not LA.

As shown in Figure 2, there were several main anomeric carbon signal peaks (δ 104.51, 102.26, and 97.65) of NMR carbon spectrum signal mainly located in δ 93–105. Among them, δ 104.51 and 102.26 were mainly β isomeric carbon, and δ 97.65 was mainly α isomeric carbon and β reductive terminal heterocarbon signal. The strong signal δ 70 also showed the existence of 6-OSO_3_-α-L-galactose. Dept135 spectrum showed that δ 68.66, 62.54, 62.37, and 62.28 were inverted peaks, indicating the chemical shift of C6, and the peak at δ 68.66 migrates to the low field, indicating the substitution of C6.

**Figure 3:**
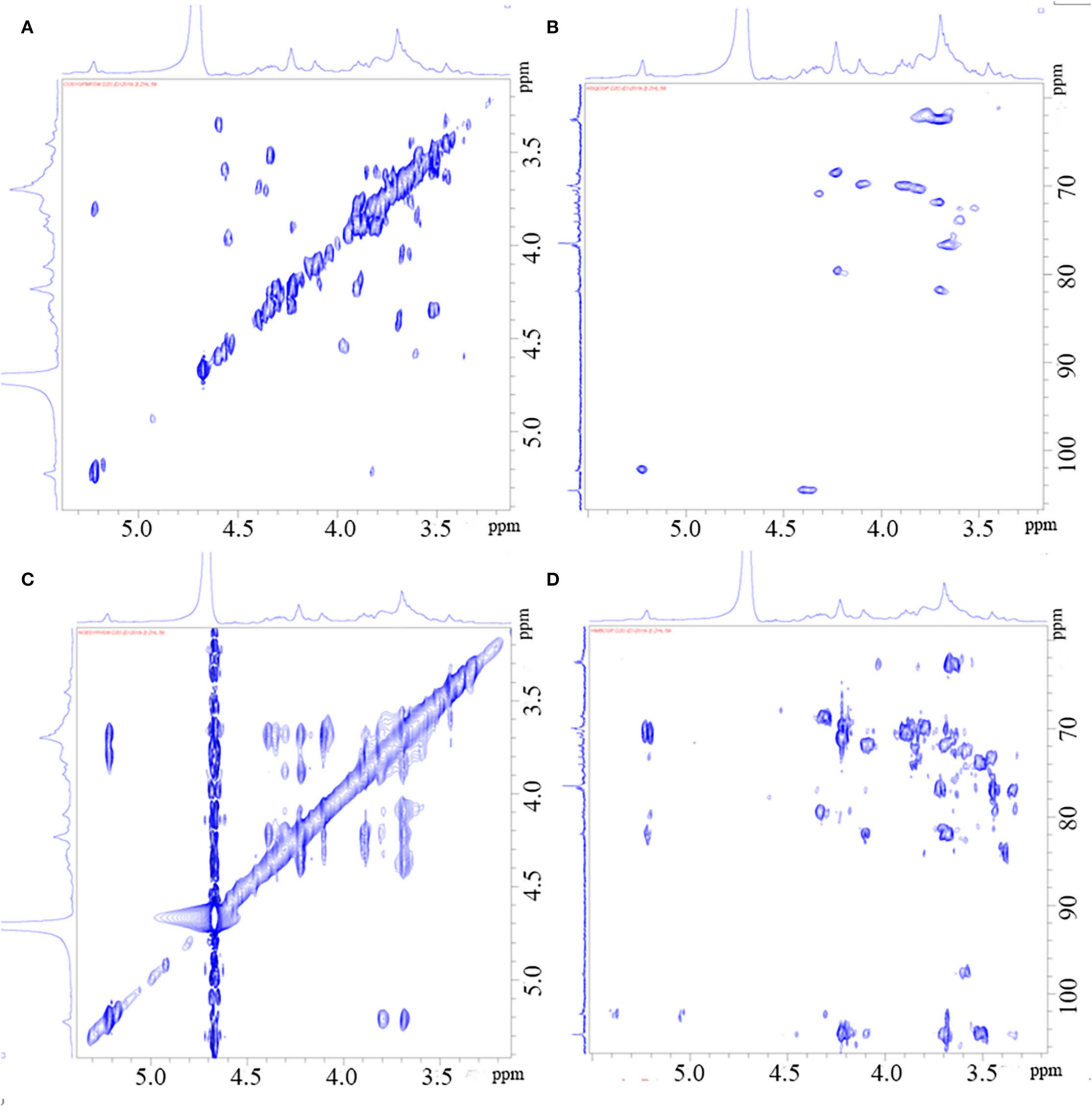
HH-Cosy spectrum of PGP **(A)**; HSQC spectrum of PGP **(B)**; NOESY spectrum of PGP **(C)**; HMBC Cosy spectrum of PGP **(D)**.

Through the HSQC spectrum, it could be observed that δ 102.4 was the heterocephalic carbon signal. The corresponding heterocephalic hydrogen signal in HSQC spectrum was δ 5.22. We could infer that H1–H5 were, respectively, δ 5.22, 3.80, 3.90, 4.23, 4.32, and 3.68. The corresponding C1–C5 is δ 102.40, 70.45, 70.05, 79.55, and 70.93. Therefore, the signal should be attributed to the glycosidic bond → 4)-α-Galp-(1 → . In the HMBC spectrum (Figure 3), we assigned the glycosidic bond signals of polysaccharides according to the one-dimensional and two-dimensional NMR spectrum. The isomeric hydrogen of → 4)-α-D-Galp has a correlation signal peak with its own C4, indicating the presence of → 4)-α-D-Galp-(1 → 4)-α-D-Galp-(1 → link mode. The isomeric hydrogen of → 3,6)-β-D-Galp-(1 → and H3 of → 3)-β-D-Galp-(1 → has a relevant signal peak, indicating the presence of → 3,6)-β-D-Galp-(1 → 3)-β-D-Galp-(1 → 3)-β-D-Galp-(1 → . The isomeric carbon of → 3)-β-D-Galp-(1 → and H4 of → 4)-α-D-Galp-(1 → has a relevant signal peak, indicating the presence of → 3)-β-D-Galp-(1 → 4)-α-D-Galp-(1 → . We combined the HMBC and NOESY spectra to assign all glycosidic bond signals, as shown in Table 2.

**Table 2:** Glycosidic bond signal attribution of PGP.

**Glycosyl residues**	**H1**	**H2**	**H3**	**H4**	**H5**	**H6a**	**H6b**
	**C1**	**C2**	**C3**	**C4**	**C5**	**C6**	
→ 4)-α-D-Galp-(1 →	5.22	3.8	3.9	4.23	4.32	3.68	ns
	102.4	70.45	70.05	79.55	70.93	62.44	
→ 3)-β-D-Galp-(1 →	4.4	3.68	3.7	4.1	3.65	4.21	ns
	104.56	71.89	81.89	69.85	76.55	68.55	
→ 3,6)-β-D-Galp-(1 →	4.4	3.68	3.7	4.1	3.65	4.21	ns
	104.56	71.89	81.89	69.85	76.55	68.55	
α-D-Galp-(1 →	5.21	3.95	3.51	3.6	3.45	3.65	ns
	102.9	70.26	72.35	72.6	74.5	62.19	

From the above results, it could be seen that PGP is a linear polysaccharide containing alternating α-(1 → 3)- and β-(1 → 4)-galactopyranose units. Most of the sulfate groups are at C6 of the -(1 → 4)-α-D-Galp, and a small part of them are etherified at C3 and C6. The methoxy signal of each type of sugar was weak, indicating that some residues were methylated. The fine structure needs to be further analyzed.

### Cell Morphology and Viability

**Figure 4:**
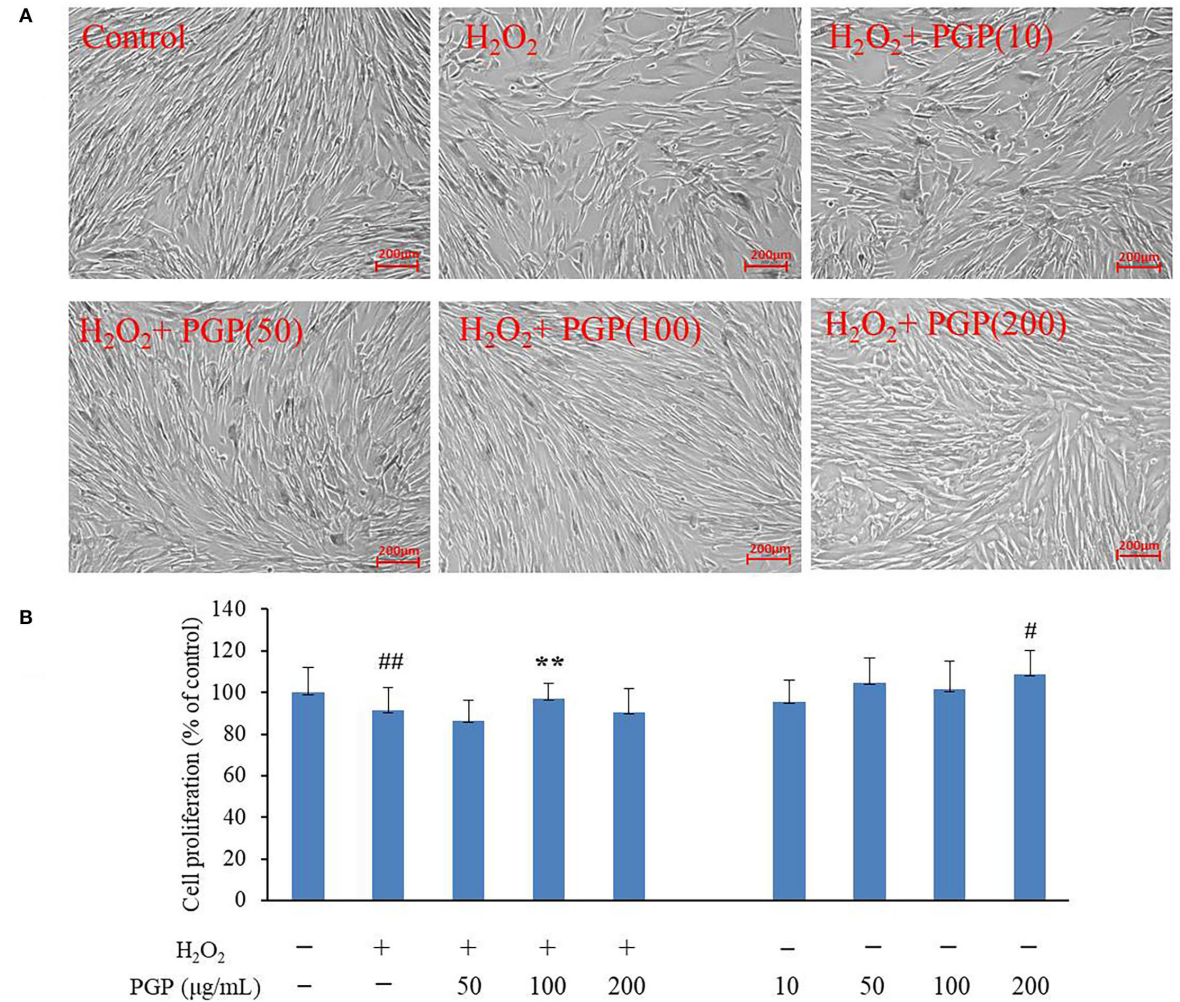
The effect of PGP on cell morphology **(A)** and cell viability in H_2_O_2_-treated young 2BS cells at PD28 **(B)** (**p < 0.01 vs. H_2_O_2_ alone group; ^##^*p* < 0.01 vs. control group). Young 2BS cells at PD28 were incubated with 200 μM H_2_O_2_ at 37°C in the culture medium for 2 h. Then, H_2_O_2_-containing medium was replaced with fresh medium containing 10, 50, 100, and 200 μg/ml of PGP for additional 72 h before being morphologically observed at the magnification of 10×. Figures are representative of three independent experiments. ^#^*p* < 0.05 vs. control group.

The MTT assay was performed to test the possible cytotoxic effects of different concentrations of PGP on cell growth. The results showed that an apparent reduction of cell proliferation was not observed upon drug treatment until the dose reached 200 μg/ml (Figure 4). It could be seen that normal young 2BS cells grow in a spindle shape and arrange regularly. They had vigorous vitality, more division phases, and clear cell boundaries. Instead, the activity of 2BS cells in the H_2_O_2_ treatment group is significantly decreased about 20%. The growth of cells is stagnant. The cell body becomes larger and the shape is flat. The arrangement is irregular, which was similar to replicative aging cells. The cytoplasmic area becomes larger, more particles or vacuoles can be seen in the cytoplasm, and the boundary is fuzzy (4). After the administration of PGP, the cells showed faint senescent morphology characteristics and displayed a fusiform appearance with oval nuclei, which could improve the cell senescence caused by H_2_O_2_ treatment in a dose-dependent manner. It was found that an exposure of 2BS cells to PGP (100 μg/ml) caused a significant livability increase of ~16% (*p* < 0.01).

### SA-β-Gal Activity

**Figure 5:**
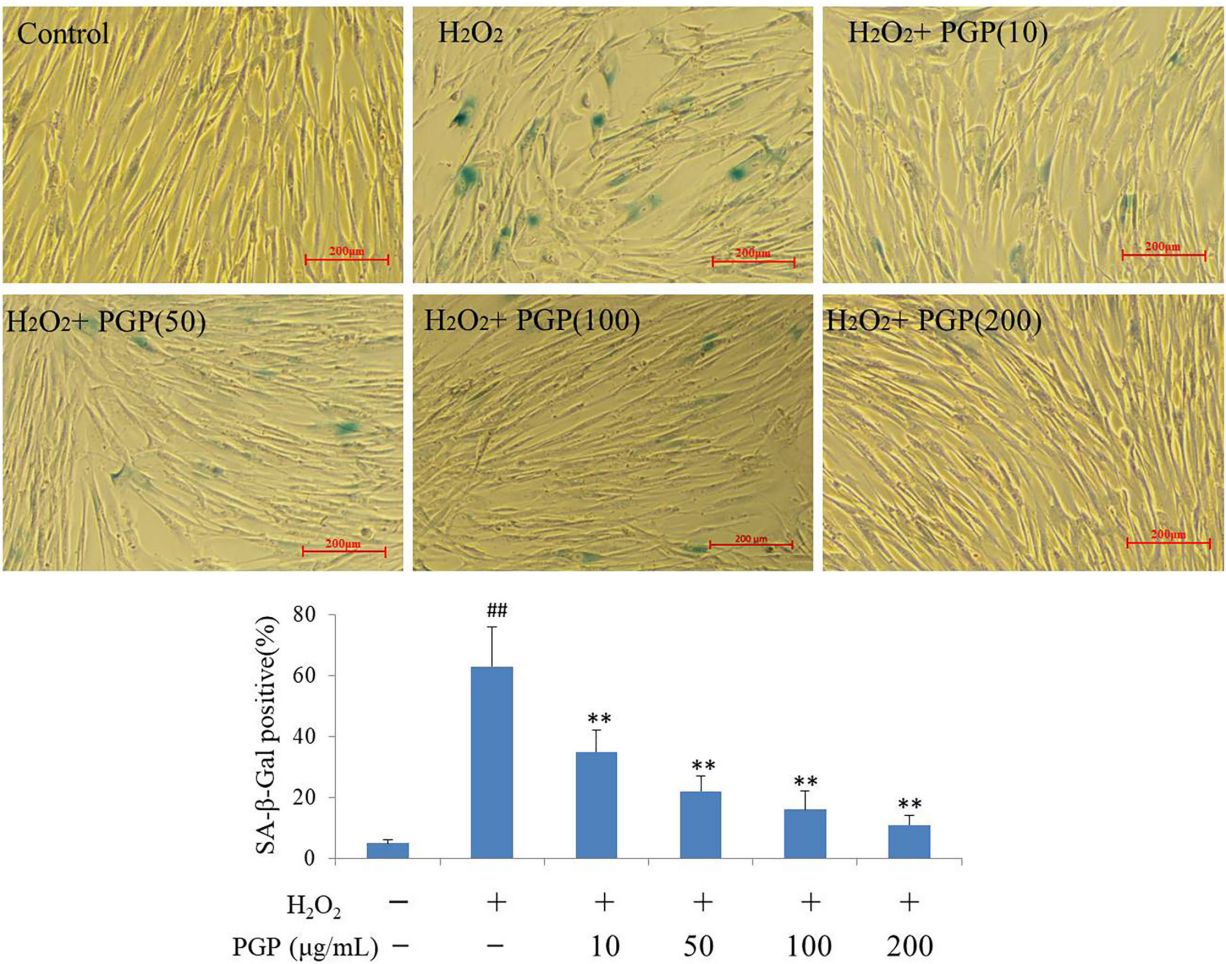
The SA-β-Gal staining of young 2BS cells treated with H_2_O_2_ followed by PGP (**p < 0.01 vs. H_2_O_2_ alone group; ##*p* < 0.01 vs. control group). PD28 2BS cells were seeded in 24-well plates for 20 h before quick exposure to H_2_O_2_ as stated above. The cells were then incubated in fresh medium without or with PGP for additional 5 days. Cells were photographed at the magnification of 10×. The percentage of SA-β-gal-positive cells out of the total cells was counted and the average data were obtained from three independent experiments.

The SA-β-gal stain is widely used to detect the signal of cellular senescence. The assay utilizes a fluorimetric substrate and cell-derived extract samples to determine quantitatively the enzymatic activity (25). As shown in Figure 5, the cells would be dyed blue-green in the process of cell replicative aging. At this time, SA-β-gal-positive rate increased gradually. The Young cells showed the lighter SA-β-Gal staining, and slender fibrous cells accounted for the vast majority. Cells in H_2_O_2_ treatment groups showed the deep SA-β-Gal staining, the increased cell volume, and the irregular shape. The SA-β-Gal-positive cells were significantly lower in cells supplemented with PGP at the concentration of 200 μg/ml (11.7%), compared to cells cultured with H_2_O_2_ (63.3%) (*p* < 0.01).

### DAPI Staining

**Figure 6:**
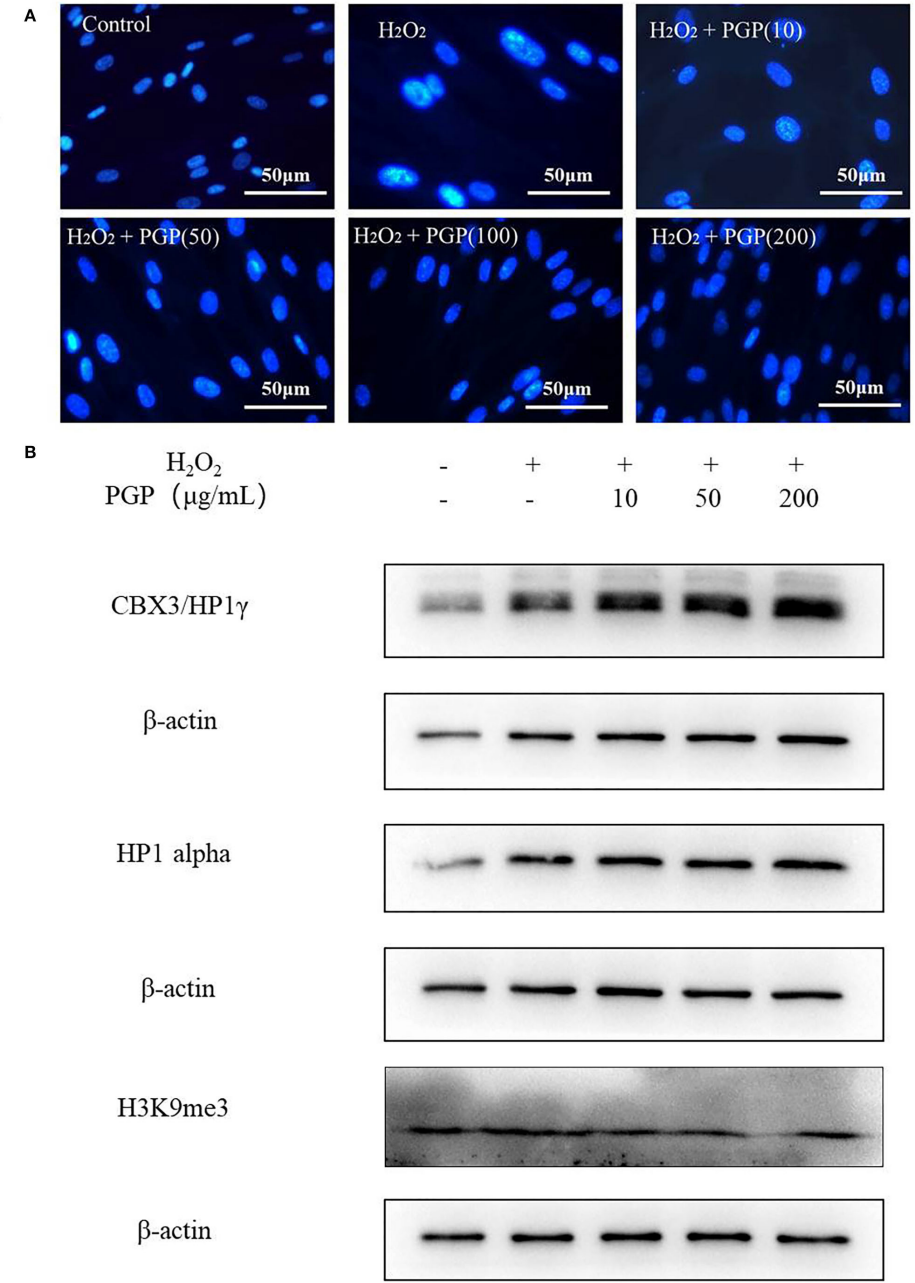
Senescence-associated heterochromatic foci (SAHF) formation **(A)** and the expression levels of three proteins (HP1a, HP1g, and H3k9me3) **(B)** in the nucleus of senescent 2BS cells visualized by DAPI staining. Permeabilize the cells by incubating the coverslips in 0.2% (v/v) Triton-X 100 in PBS (pH 7.3) for 5 min at room temperature. Stain SAHF by incubating the coverslips with 0.15 μg/ml final concentration of DAPI diluted in 3% (w/v) BSA in PBS, pH 7.3 for 3 min. After the slides have dried, observe SAHF using a fluorescent microscope.

At the chromatin level, the occurrence of cell senescence is often accompanied by extensive changes in chromatin structure, with dot-like aggregation of heterochromatin structure and inhibition of the expression of cell proliferation-related genes. This characteristic phenomenon is called senescence-associated heterochromatic foci (SAHF) (26). Due to the consistent localization of HP1, H3K9me2/3, and SAHF, they have become the marker proteins of aging-related heterochromatin aggregation. This provides a method for researchers to judge whether cells have entered the aging state, that is, to observe the distribution of HP1 (or H3K9me2/3) and chromatin in the nucleus at the same time by the immunofluorescence method (27). From Figure 6, after DAPI staining, the cells in the H_2_O_2_ treatment group showed an obvious aging state with enlarged nucleoli compared with the control group. This indicated that the cells had entered the aging state. PGP pretreatment could prevent the formation of SAHF induced by H_2_O_2_ in a dose-dependent manner. When the doses were increased to 100 and 200 μg/ml, SAHF was significantly reduced to the level close to the control group. Western blotting showed that the expression levels of three proteins (i.e., HP1α, HP1γ, and H3k9me3) were upregulated in the model group. The sample PGP could downregulated the marker protein. These results clearly indicated that PGP was able to influence the cell cycle, leading to improved cell proliferation and delaying premature senescence induced by H_2_O_2_.

### The Expression of Senescence-Associated Molecular Markers

**Figure 7:**
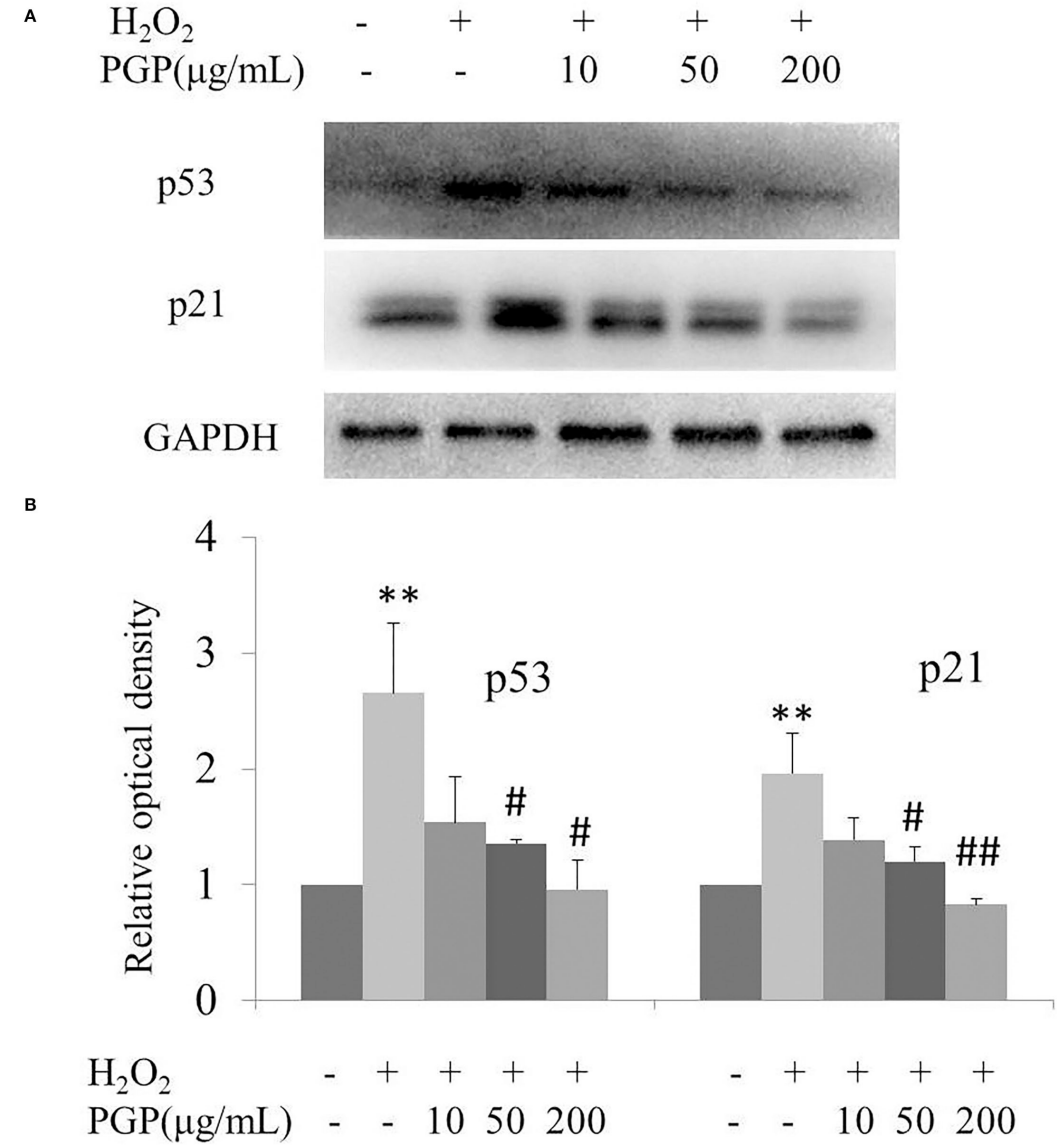
The expression of senescence-associated proteins **(A)** and relative optical density of P53 and P21 in H_2_O_2_-treated young 2BS cells incubated with various concentrations of PGP (**p < 0.01 vs. control group; ^#^*p* < 0.05 vs. H_2_O_2_ alone group; ^*##*^*p* < 0.01 vs. H_2_O_2_ alone group). Bars represent relative protein levels counted as *D*1*/D*0 (the value for control was set as 1.0), where *D*0 and *D*1 stand for the optical density of GAPDH ladder and sample ladder, respectively. The optical density for each ladder was calculated using the ImageJ software. Data were obtained from three independent experiments.

The results of this experiment showed that the intracellular p53 and p21 of 2BS at the protein level were significantly increased after H_2_O_2_ treatment. PGP could decrease the expression of p53 and p21 in a dose-dependent manner, of which the most significant downregulated levels of p53 and p21 were treated with the concentration of 200 μg/ml (Figure 7). It was speculated that PGP may delay aging by downregulating the expression of p21 and p53.

## Discussion

Acid extraction is performed to obtain polysaccharides with dilute hydrochloric acid or rare organic acid as solvent. The activity was better, and the yield of polysaccharides is in high quantity (28). However, the glycosidic bond is easy to break under acidic conditions, which will affect the activity and structure of polysaccharides, but some novel polysaccharide chains can be obtained to increase the diversity of polysaccharide structure. The outer epidermis of *G*. *lemaneiformis* is hard which is difficult to obtain a higher yield of polysaccharides. Therefore, searching for ways to be more efficient to extract polysaccharides had become the investigations of many researchers. At present, solvent (e.g., water) extraction, physical-assisted (ultrasonic, microwave, etc.) extraction, and enzymatic hydrolysis extraction had become the main extraction methods of GP (29). There were many related reports before, but there was a less in-depth exploration of structural analysis. The polysaccharide obtained by *G*. *lemaneiformis* with citric acid at a lower concentration had been reported, which had a high molecular weight (30). In our study, the concentration of citric acid was increased to obtain polysaccharides with lower molecular weight. From the results, the expected polysaccharide with lower molecular weight was obtained. After separation and purification, we obtained the polysaccharide with good water solubility. The isolation of this sulfated polysaccharide from *G*. *lemaneiformis* added a new type of polysaccharide to this group of red seaweeds.

Senescent cells have become an emerging target for the disease of aging (31). H_2_O_2_ was known to induce cellular senescence showing multiple changes. Based on this study, we have employed low-passage cells and the appropriate H_2_O_2_ concentration to induce premature senescence was determined to be 200 mM. Senescent cells acquire a typical flat and enlarged shape and show the increased levels of a group of senescence biomarkers, including SA-β-Gal activity, and the protein abundance of p21 and p53 (32, 33). Studies suggested that the long-term supplementation of SHQA also notably delayed the growth arrest and lessened the raise of senescence biomarkers, including p53, p21, and SA-β-Gal. Consistently, we observed that upon exposure to low-dose H_2_O_2_, the abundance of SA-β-Gal-positive cells was elevated in 2BS; meanwhile, the pretreatment of PGP significantly suppressed their elevations, which suggested the ability of PGP to repress the oxidative stress-induced senescence in 2BS.

The p53-p21 pathway is a relatively recognized aging-related signal pathway. Inactivation of p53 or p21 gene can prolong the replicative life of cells, indicating that this signal pathway is closely related to cell aging (34). p53 and p21 are cell cycle regulators directly related to aging (35). When cells encounter some external stimuli, it is found that the expression of p53 will be rapidly upregulated with stress, so as to further activate p21, inhibit the phosphorylation of p21 protein, block the normal cell cycle, and then lead to cell aging. The target molecule of p53 is p21, which is a cyclin-dependent protein kinase inhibitor that inhibits cell cycle and can make cells enter an irreversible growth arrest state (36). In this study, the partial inhibitory effects of PGP on 2BS may be due to the contribution of the p53-p21 pathway.

## Conclusion

The polysaccharides isolated from *G*. *lemaneiformis* were slightly methylated agarans, with a low 3,6-anhydrogalactose content, bearing sulfated mainly at the 6-position of (1 → 4)-α-D-Galp. Our studies demonstrated that H_2_O_2_ caused cell cycle arrest accounting for inhibition of cell proliferation, resulting in SA-β-gal activity elevation and SAHF formation. Pretreatment with PGP protected against H_2_O_2_-induced cellular senescence. The anti-aging effect of PGP may be involved in potentiating the p53-p21 pathway. These results suggested the promising role of PGP as an attractive food with the potential to retard senescence and senescence-related diseases.

## Data Availability Statement

The original contributions presented in the study are included in the article/supplementary material, further inquiries can be directed to the corresponding author/s.

## Author Contributions

XW: visualization, investigation, and validation. XX and GM: feeding cells and supporting partial data. GW and XS: methodology, data analysis, drawing charts, and writing—original and editing. YG: conceptualization, methodology, and writing —review. NX: investigation and formal analysis. ZZ: funding acquisition and supervision. All authors have read and agreed to the publishing of the current version of the manuscript.

## Funding

This study was supported by the Zhejiang Province Basic public welfare research project (LGN21D060001) and the National Natural Science Foundation of China (31700307 and 81771520).

## Conflict of Interest

The authors declare that the research was conducted in the absence of any commercial or financial relationships that could be construed as a potential conflict of interest.

## Publisher's Note

All claims expressed in this article are solely those of the authors and do not necessarily represent those of their affiliated organizations, or those of the publisher, the editors and the reviewers. Any product that may be evaluated in this article, or claim that may be made by its manufacturer, is not guaranteed or endorsed by the publisher.
